# Efficacy and Safety of *Boswellia serrata* and *Apium graveolens* L. Extract Against Knee Osteoarthritis and Cartilage Degeneration: A Randomized, Double-blind, Multicenter, Placebo-Controlled Clinical Trial

**DOI:** 10.1007/s11095-025-03818-2

**Published:** 2025-01-28

**Authors:** Narendra Vaidya, Ramshyam Agarwal, D. G. Dipankar, Hrishikesh Patkar, Gayatri Ganu, Dheeraj Nagore, Chhaya Godse, Anirudh Mehta, Dilip Mehta, Sujit Nair

**Affiliations:** 1 Lokmanya Medical Research Center and Hospital, Pune, 411033 India; 2 Dr. D. Y. Patil College of Ayurved & Research Centre, Pimpri-Chinchwad, 411018 India; 3 Sangvi Multispeciality Hospital Pvt. Ltd, Pune, 411027 India; 4 MPREX Healthcare Pvt. Ltd., Pune, 411057 India; 5 Phytoveda Pvt. Ltd., V.N. Purav Marg, Mumbai, 400022 India; 6 Viridis Biopharma Pvt. Ltd., Mumbai, 400022 India

**Keywords:** cartilage support, clinical trial, inflammation, knee osteoarthritis, nutraceutical

## Abstract

**Background:**

Osteoarthritis is the prevailing form of inflammatory condition in joints of adults and the aging population, leading to long-term disability and chronic pain. Current therapeutic options have variable therapeutic efficacy and/or several side effects.

**Methods:**

A randomized, placebo-controlled, double-blind clinical trial was conducted in 62 participants using a nutraceutical [standardized *Boswellia serrata* Roxb. gum resin (300 mg) and *Apium graveolens* L. seed extract (250 mg)], to determine its safety and efficacy for supporting cartilage health and reduction in knee osteoarthritis symptoms. All participants were assessed for physical function and pain with the help of WOMAC, VAS, Physicians' Global Assessment for the six-minute walk test/pain. Knee X-ray, KOOS questionnaire score, and FACIT-F score were assessed. Additionally, inflammatory, cartilage degeneration and regeneration biomarkers in serum and urine were evaluated at baseline and after 90 days of treatment.

**Results:**

Oral administration of the nutraceutical resulted in prolonged symptomatic relief with reduced pain, stiffness, and swelling. Inflammatory (serum IL-7, IL-1, IL-6, hs-CRP, TNF-α, ESR) and cartilage degeneration biomarkers (serum CTX-II, COMP, MMP-3 and urinary CTX-II) were decreased in the nutraceutical group compared to baseline and placebo. Furthermore, serum N-propeptide of collagen IIA (PIIANP) and procollagen-type-C propeptide (PIICP) levels were increased in the nutraceutical group, suggesting collagen synthesis contributing to cartilage regeneration. At given doses for 90 days, there were no adverse effects based on the clinical examination, biochemical, hematological, and ECG analysis.

**Conclusions:**

Taken together, the combination of Boswellia and celery could be a safe and promising herbal nutraceutical option for managing osteoarthritis and cartilage health effectively.

**Supplementary Information:**

The online version contains supplementary material available at 10.1007/s11095-025-03818-2.

## Introduction

Osteoarthritis (OA) is a common degenerative disease characterized by swelling, pain, mobility loss and stiffness. It affects the knees, hands and hips with more postmenopausal women (18%) than men (9.6%) suffering from OA [1]. In addition, OA affects ligaments, synovium, and subchondral bone, and significantly increases the burden of disability worldwide [1]. Currently, 58 million adults are suffering with osteoarthritis and by 2040, this number is anticipated to grow to 78.4 million as it is the primary cause of pain in the joints and functional limitations worldwide [2]. Osteoarthritis affected 595 million persons worldwide in 2020, accounting for 7.6% of the world's population and a 132.2% rise in cases since 1990; by 2052, it is anticipated that cases of osteoarthritis will rise by 74.9% for the knee, 78.6% for the hip, 48.6% for the hand and 95.1% for other types of arthritis compared to 2020 [3, 4].

At present, there are no medications available that can prevent or slow the progression of osteoarthritis. Pain control, preserving or enhancing quality of life and joint mobility are the key goals of pharmacologic therapy, in addition to non-pharmacologic treatments like weight loss, physical therapy and exercise. Although oral NSAIDs have been shown to be beneficial for the treatment of osteoarthritis (OA), their usage is restricted to particular patient categories due to their considerable side effects and contraindications. Higher risk of cardiovascular events, decreased kidney function, gastrointestinal bleeding, and fluid retention are among the most severe side effects [5]. Patients with severe OA, accompanied by articular cartilage damage, may require intra-articular injections of hyaluronic acid or glucocorticoids. However, glucocorticoids adversely affect bone and cartilage by developing lesions at articular cartilage and subchondral bone that eventually cause bone loss. Hyaluronic acid can have multiple benefits, as it restores the viscoelasticity of joint fluid, improves lubrication and shock absorption, reduces joint friction, and relieves pain. However, it must be injected intra-articularly in a joint cavity, requiring expertise and an operation set-up. It may cause short-term side effects such as joint edema and infection, and controversies about its variable efficacy exist [6]. Several nutraceuticals and dietary supplements have been claimed to be effective in one or more osteoarthritic pathogenic processes [6]. Glucosamine and chondroitin sulphate, methyl sulfonyl methane (MSM), collagen, omega-3 fatty acids, vitamins, and various plant-derived products have been studied clinically and shown to be effective [7, 8]. However, the results are variable, and their efficacy and safety are controversial. Clinical evidence through meta-analysis studies shows limited benefits through symptomatic relief with marginal and inconsistent outcomes [9]. Moreover, the effects on pathogenesis, cartilage, or bone health are not demonstrated. As per the Cochrane review of 25 clinical studies (4963 patients), glucosamine failed to show any benefit, and its effect on pain was as good as that of a placebo [10]. The American College of Rheumatology and the Arthritis Foundation both prescribe chondroitin sulphate for OA, in accordance with the 2019 therapy guidelines. The National Center for Complementary and Integrative Health (NCCIH) asserts that chondroitin is inefficient and that the data regarding glucosamine's potential to relieve OA pain is ambiguous [11]. As a result, there is a continuous need for new and safe therapeutic options for the management of OA. Recently, the use of mesenchymal stromal cell therapy has been explored for treating osteoarthritis [12]. Medicinal plants and their constituents are also being explored for this unmet need. In Traditional Chinese Medicine (TCM), the efficacy and safety of Gutong Patch has been compared with NSAIDs for knee osteoarthritis [13] and Wuwei Shexiang pill has been reported to show anti-arthritic effects [14]. The *Boswellia serrata* tree is prevalent in India and its gum, or guggul, has been shown to have good analgesic, anti-inflammatory, and anti-arthritic properties. *B. serrata c*ontains β-configured pentacyclic triterpene acids, which are significant components of the extract. These include 11-keto-β‐boswellic acid (KBBA), 3- acetyl-11-keto-β‐boswellic acid (AKBBA), 3‐acetyl‐β‐boswellic acid (ABBA) and β‐boswellic acid (BBA) [15]. There is 2% volatile oil in celery seed. About 60% and 20% of the oil consists of limonene and selinene, respectively. However, 3-n-butyl phthalide, 3-n-butyl-4–5-dihydrophthalide (sedanenolide), sedanonic anhydride and sedanolide —all of which are found in inadequate amounts (1–3%) —are the primary flavor ingredients of the oil giving it its characteristic aroma [16]. Celery seed extract is at least as effective as ibuprofen, aspirin and naproxen in suppressing arthritis in a model of polyarthritis by decreasing inflammation in rats and providing an analgesic effect [17].

The present study was intended to examine the safety and efficacy of standardized *Boswellia serrata* and *Apium graveolens* L. seed extract against knee osteoarthritis and cartilage degeneration. To the best of our knowledge, this is the first study using a combination of *Boswellia serrata* oleo gum resin and *Apium graveolens* L. seed extract, conducted for 90 days + 30 days (after stopping therapy) in knee osteoarthritis patients by measuring standard parameters, radiography, cartilage degeneration and regeneration biomarkers to assess the efficacy of the nutraceutical. Furthermore, product safety and tolerability were well established by evaluating the hematological, biochemical and ECG analysis at baseline and upon completion of the clinical trial in all the participants. This study evaluates the safety and efficacy of the combination of Boswellia and celery in human patients.

## Material and Methods

### Study Design and Objectives

**Fig. 1:**
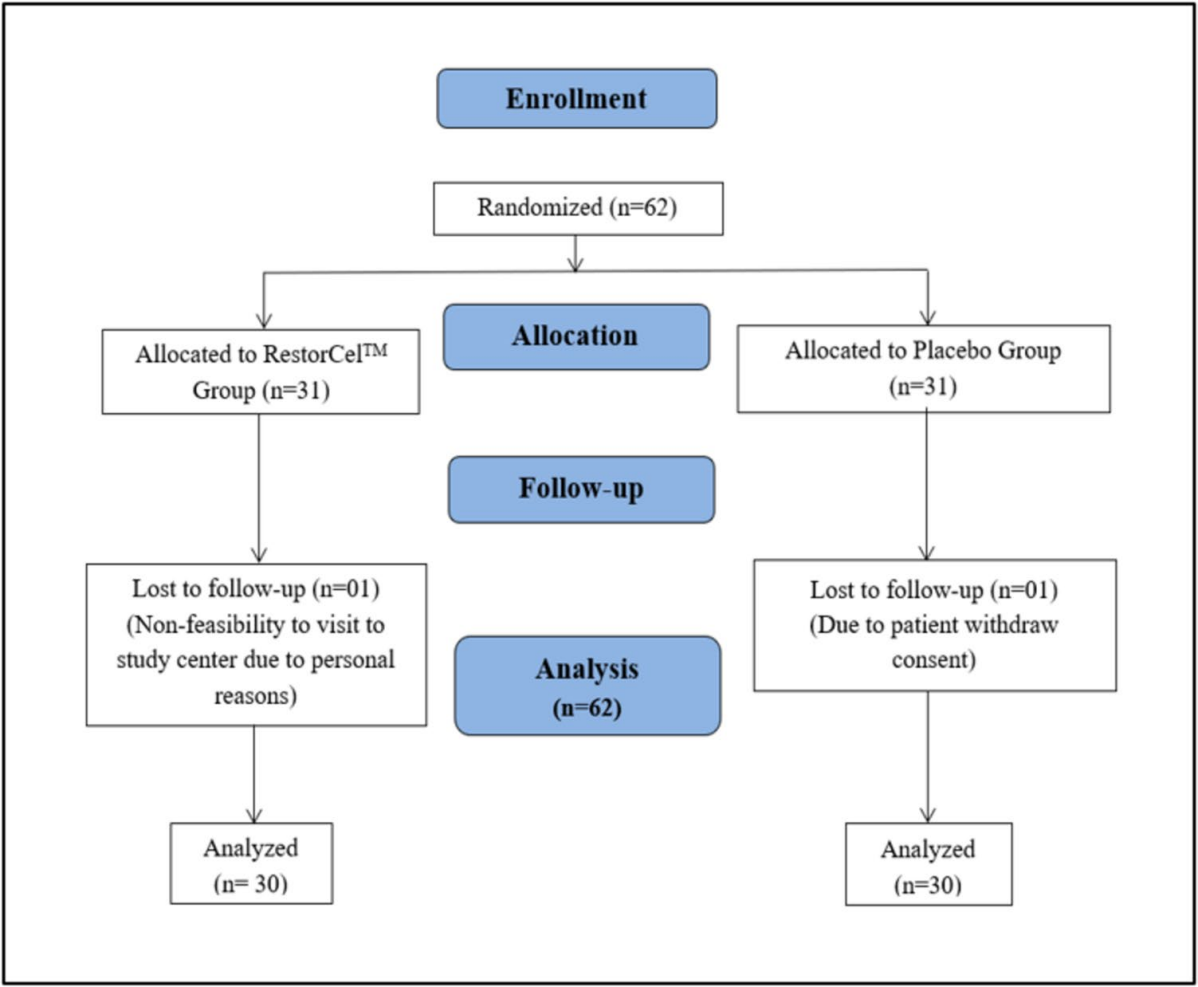
Study Protocol: Patients were instructed to consume the designated capsule (nutraceutical or placebo) twice a day, 30 min after breakfast and dinner for 90 days.

A randomized, multicenter, double-blind, placebo-controlled, parallel-group clinical trial was conducted at three sites: Lokmanya Medical Research Centre and Hospital, Sangvi Multispecialty Hospital Pvt. Ltd., and Dr. D. Y. Patil College of Ayurved and Research Centre, Pune, Maharashtra, India. The study design is depicted in Fig. 1.

### Investigational Product

RestorCel™ is a nutraceutical product which is a combination of *Boswellia serrata* (300 mg) + *Apium graveolens* L. (celery) seed extract (250 mg) marketed by Phytoveda Pvt. Ltd., Mumbai, India which funded this study. Thus, RestorCel™ is a standardized blend (550 mg) containing extracts from *Boswellia serrata* gum and *Apium graveolens* L. seeds, 300 and 250 mg, respectively. Figure S1 represents the measured components of RestorCel™ using HPLC–PDA. For Boswellia serrata, the phytopharmaceutical actives were quantified by using HPLC–PDA detector. These analytes were separated using a C18, 25 cm × 4.6 mm, 5 µ analytical column. Water acidified with ortho phosphoric acid and acetonitrile was used as a gradient mobile phase. The analytes were monitored at 210 nm and 250 nm based on their maximum response for UV detector. The analytes were separated over a period of 45 min. Analytes were quantified against their reference standards. For Apium graveolens, the phytopharmaceutical actives were quantified by using HPLC–UV detector. These analytes were separated using a C18, 25 cm × 4.6 mm, 5 µ analytical column. Water acidified with formic acid and acetonitrile was used as a gradient mobile phase. The analytes were monitored at 330 nm based on their maximum response for UV detector. The analytes were separated over the period of 50 min. Analytes were quantified against their reference standards. Table I depicts the compounds identified in each capsule by HPLC–PDA. The extract is prepared in veggie capsules (Size '00') with a dosage of 550 mg and manufactured in a state-of-the-art GMP-certified facility. The placebo capsule contained an equal amount of Dextrin.

**Table I:** Compounds Identified in Each Capsule by HPLC–PDA

Compound	Content (mg/capsule)										
	CAP 1	CAP 2	CAP 3	CAP 4	CAP 5	CAP 6	CAP 7	CAP 8	CAP 9	CAP 10	MEAN ± SD
11-keto-B-boswellic acid	29.27	29.96	29.59	31.67	31.29	31.79	29.51	27.66	28.96	29.48	29.92 ± 1.30
3-acetyl-11-keto-B-boswellic acid	3.52	3.53	3.71	3.92	3.74	3.73	3.42	3.59	3.48	3.65	3.63 ± 0.15
Serratol	19.24	19.67	19.91	21.04	20.88	21.21	19.50	18.31	19.37	19.55	19.87 ± 0.92
3-O-keto-tirucallic acid	18.49	18.91	18.88	20.12	19.97	20.25	18.67	17.55	18.43	18.72	19.00 ± 0.86
A-Boswellic acid	20.96	21.34	21.22	22.53	22.22	22.78	21.13	19.73	20.67	21.22	21.38 ± 0.91
3-O-acetyl-A-tirucallic acid	2.35	2.38	2.30	2.43	2.40	2.56	2.39	2.19	2.31	2.33	2.36 ± 0.10
B-Boswellic acid	56.19	57.36	56.49	59.83	57.74	60.86	56.66	52.78	55.44	56.47	56.98 ± 2.24
3-O-acetyl-B-tirucallic acid	0.12	0.11	0.12	0.12	0.11	0.12	0.11	0.12	0.11	0.11	0.11 ± 0.01
3-acetyl-A-boswellic acid	4.38	4.27	4.35	4.75	4.53	4.60	4.39	4.28	4.20	4.20	4.40 ± 0.18
3-acetyl-B-boswellic acid	15.54	16.02	15.77	17.94	16.98	16.44	17.15	15.88	16.49	16.95	16.52 ± 0.75
Apiin	1.59	1.54	1.52	1.48	1.44	1.59	1.42	1.40	1.47	1.42	1.49 ± 0.07
Bergapten	4.97	4.77	5.07	4.77	5.17	4.92	4.77	4.87	5.12	4.63	4.91 ± 0.18
Seselin	29.78	28.52	30.43	28.60	31.09	29.46	28.22	27.26	28.58	27.66	28.96 ± 1.21

#### Manufacturing and Quality Control of Investigational Product

The gum resin of *Boswellia serrata* and seed of *Apium graveolens* L. were freshly sourced and authenticated. Both materials were separately subjected to the pretreatment and extraction process. The dried material was extracted by reflux extraction with hydroalcoholic solvent. The extract layer was filtered through filter aids under vacuum pressure. The cycles were repeated until the complete extraction of targeted bio-actives had been achieved. The combined reflux pooled extracts were concentrated to reduce the volume. The residue was milled and sieved to get a powder extract. The physical parameters (identification, description, colour, odour, taste, and foreign matter), heavy metal and microbial analysis were performed for both extracts as per USP specifications [18–22]. After analysis, the powdered extract without any excipient was encapsulated in the GMP-Certified facility in a veggie capsule size “00” containing 300 mg *Boswellia serrata* and 250 mg celery seed extracts.

#### Assessment Methods

**Table:** 

Chemical Analysis	
Total Boswellic Acids	NLT 120.0 mg/capsule
Total content (Total Boswellic Acids + Total tirucallic acid + Serratol)	NLT 150.0 mg/capsule
Total content Apium graveolens extract actives (Apiin + Bergapten + Seselin)	NLT 15.0 mg/capsule
Disintegration Time	NMT 30 min
Weight Variation	720 mg ± 7.5%
Impurities	Lead: < 0.50 ppm Mercury: < 0.50 ppm Arsenic: < 1.00 ppm Cadmium: < 0.50 ppm Residual solvents: Meets USP-NF < 467 >
Microbial Analysis	Total plate count: < 1000 cfu/g Yeast & Mold: < 100 cfu/g Coliform: < 3.0 mpn/g Enterobacterial Count: < 100.00 cfu/g E. coli: Absent Salmonella: Absent Staphylococcus aureus: Absent

### Objectives of the Study

The study aimed to assess the efficacy and safety of nutraceutical, administered orally, in managing knee osteoarthritis. The primary objective of the study was to determine the effect of RestorCel™ on the symptoms of osteoarthritis, viz. pain, stiffness, and mobility, in OA patients. The secondary objectives of this study were to determine changes in inflammatory markers and dependency on analgesic medication and confirm the product's safety by monitoring adverse events.

### Ethics Approval and Registration of Clinical Trial

The Drug and Cosmetic Act of 1940, the Drug and Cosmetic Rules of 1945, the World Medical Association's (WMA) Declaration of Helsinki, the International Conference on Harmonization's (ICH) recommended harmonized tripartite guideline regarding Good Clinical Practice, and other ethical principles and guidelines were all followed during the conduct of the clinical trial (ICH-GCP). Institutional Ethics Committees (IECs) of Lokmanya Medical Research Centre & Hospital and Sangvi Multispecialty Hospital Pvt. Ltd., Pune, Maharashtra, India has approved the study. The clinical trial and protocol were registered with the Clinical Trial Registry-India (CTRI) with approval number CTRI/2023/07/054799. Throughout the investigation, the authorized clinical procedure did not alter.

### Informed Consent

Thorough explanation of the design, expected benefits and potential hazards of the study was received by patients before participating. The participants could understand the format and language in which this information was given. Participants were informed that they could leave at any time during the study. Each participant gave written consent before being enrolled in the study. Participants were informed about the objectives, methods, possible risks and benefits of the study and confidentiality policies, and the institutional ethics committee's and study investigator's contact details. To maintain participant confidentiality, all personal data and study information were tagged and securely archived. Anonymity of the participants was preserved in all publications and study reports. The data were only accessible to approved study personnel.

### Study Participants

Sixty-two patients were recruited in a double-blind, randomized, placebo-controlled multicenter clinical trial of *Boswellia serrata* and *Apium graveolens* L. (Celery) seed extract for knee osteoarthritis management. This double-blind, placebo-controlled clinical trial evaluated a nutraceutical combining *Boswellia serrata* gum and *Apium graveolens* L. seed extracts in mild to moderate OA. The criteria listed below were used to select participants.

#### Inclusion Criteria

In this study, adult women and men of ages 40—65 were included with a BMI < 30.00 kg/m^2^. Individuals who met the criteria set by the American College of Rheumatology (ACR) for clinically confirmed diagnosis of knee osteoarthritis were included in the study. Knee pain accompanied by at least three out of six symptoms: bony tenderness, crepitus, bony enlargement, and absence of palpable warmth age > 50 years, morning stiffness < 30 min, were included in the study. In the 24 h prior to recruitment, study participants who were enrolled in the trial had a minimum pain VAS score of more than four when walking in one or both knees. The study included ambulant individuals who were in need of anti-inflammatory drug treatment, were not regularly taking anti-inflammatory or analgesic medications, or who were unhappy with their present prescription and desired to change. Before beginning the study intervention, participants also had to agree to go through a washout period for analgesics that lasted at least three days. They also had to commit to show up for follow-up appointments on a regular basis. The study comprised walkable participants who could provide written and spoken information about the investigation.

#### Exclusion Criteria

Individuals with a known record of hypersensitivity to herbal extracts or dietary supplements were excluded from the study. Women who were nursing or pregnant, those who were not using sufficient forms of birth control, and those who tested positive for urine pregnancy test were also not included. Participants with non-degenerative joint diseases or other musculoskeletal disorders that could interfere with OA evaluation, such as active gout, rheumatoid arthritis, joint infection or recent joint trauma, were not included in the study. Additionally, persons who couldn't perform self-care tasks because they were disabled or confined to a wheelchair or bed were also not included. Participants undergoing treatment with hydantoin, anticoagulants, colchicine, lithium, steroids, or methotrexate, were not eligible. Participants with evidence of severe hepatic, renal, or hematopoietic diseases, or severe cardiac insufficiency were excluded based on specific criteria and based on the clinical investigators' judgement. Severe hepatic or renal diseases were defined as laboratory values exceeding 1.5X the upper limit of normal LFT, and RFT. Additionally, baseline assessments required CBC values to fall within normal ranges and no significant alterations in ECG findings. Individuals who had received Ayurvedic formulations or Complementary and Alternative Medicine (CAM) therapy in the preceding three months were excluded. Hyaluronic acid administration over the previous nine months and intra-articular steroid administration within the previous three months were also not included in the study. Exclusion from the study occurred if the participant had an arthroscopy of either knee or surgery for osteoarthritis in the previous year. The subject was also eliminated from the study if, in the investigator's judgment, their condition failed to justify their inclusion.

### Clinical Study Procedure

In this study, participants taking analgesics were enrolled after an appropriate washout period of at least three days. Two groups of 62 participants each were randomly assigned to receive either nutraceutical [*Boswellia serrata* (300 mg) + *Apium graveolens* L. (celery) seed extract (250 mg)] or a placebo capsule in a 1:1 ratio (31 people in each group) (Fig. 1). The treatment duration was 90 days, and 120-day study period. The efficacy of the investigational products was compared between the groups. Concomitant diseases/medication assessment was performed on screening. Patients were instructed to consume the designated capsule twice daily 30 min after breakfast and dinner for 90 days. The dose of nutraceutical was estimated based on the data from preclinical *in vivo* studies for safety and efficacy and from a pilot clinical study at doses of 275 and 550 mg two times for the duration of 90 days (unpublished data). The following medications were not permitted during the study: Ayurveda, homoeopathy, Siddha, Unani, and nutraceutical supplements for knee osteoarthritis. The other rescue medication was allowed at the discretion of the investigator, but was documented in the case report form (CRF). Celecoxib (100 mg bid) SOS was given to the participants as analgesic rescue medication at the investigator's discretion during the study and recorded in the case record form. Treatment for other concomitant diseases was continued at the discretion of the investigator. Medication permitted as per the discretion of the investigator for the same was also noted in the CRF. Urine samples were collected for the quantification of urine CTX-II levels. A trained phlebotomist collected blood samples at each study site during the screening and day 90 visits, with each sample having a maximum volume of 10 ml. For safety assessment, 5 mL of blood was collected and divided into 2 mL in an EDTA tube and 3 ml in a plain tube, while for biomarker assessment, 5 ml was collected in a gel tube. A centralized NABL-accredited laboratory (My Labs Healthcare, Office No 15, 1st Floor, Ganesham Commercial, Sai Nagar Park, Pimple Saudagar, Pune, Pimpri-Chinchwad, Maharashtra 411027) processed the safety assessment of samples. The samples for biomarker assessment were then stored in a deep freezer at −80°C ± 10°C until the completion of participants' enrollment. The quantitative measurement of overall serum PIIANP, Human Procollagen 2-C-terminal Peptide, COMP, CTX-II, TNF-α, IL-7, IL-1, IL-6, hs-CRP, MMP 3 levels was performed using a multiplex Enzymatic immunoassay (ELISA) assay. The kits were manufactured by Krishgen Biosystems, Mumbai, India. Biomarker analysis was conducted at the Bioanalytical Facility of the Dr. D. Y. Patil Institute of Pharmaceutical Sciences & Research, Department of Pharmaceutical Chemistry, Pune, India. The p-values were corrected for multiplicity using a post hoc Bonferroni correction.

### Follow-Up and Monitoring

Follow-up study visits were scheduled for the participants as baseline (day 0) and on days 1^st^, 3^rd^, 7^th^, 15^th^, 30^th^, 45^th^, 60^th^ and 90^th^. Patients underwent clinical, systemic and safety examinations at every visit.

### Clinical Outcome Measures

The study's primary efficacy measure was to compare improvements in WOMAC A, B, and C subscale scores for pain, stiffness, and physical impairment at screening days 7th, 15th, 30th, 45th, 60th, and 90th. WOMAC-A was evaluated using a Likert scale ranging from 0 (none) to 4 (severe). Higher scores corresponded to increased levels of pain for WOMAC-A whereas WOMAC-B was used for stiffness levels and WOMAC-C for physical disability. The WOMAC and other endpoints were assessed by investigators at all three sites. To minimize inter-rater variability, an investigator meeting was conducted to ensure consistency in assessments across all sites. Changes in the physician’s global assessment for pain were carried out at following screening days: 1st, 3rd, 7th, 15th, 30th, 45th, 60th, and 90th. The differences in distance covered during the six-minute walk test were evaluated on the following days 0, 7th, 15th, 30th, 45th, 60th, and 90th respectively. Changes in VAS pain scale score were evaluated at following screening days 1st, 3rd, 7th, 15th, 30th, 45th, 60th, 90th, 105th and 120th respectively. Furthermore, radiological examinations (X-rays) were carried out on all participants on day 0 and day 90, respectively (Table II).

**Table II:** Assessment Schedule for Clinical Evaluation and Investigations

Assessment	Days of Evaluation
WOMAC Subscale Scores (A, B, C) & GI Symptoms	Screening, 7, 15, 30, 45, 60, 90
Physician's Global Assessment (Likert Scale) and and OA-related symptoms on the Likert scale	Screening, 1, 3, 7, 15, 30, 45, 60, 90
6-Minute Walk Test	0, 7, 15, 30, 45, 60, 90
VAS Pain Score	1, 3, 7, 15, 30, 45, 60, 90, 105 (telephonic), 120 (telephonic)
Biomarker levels (serum and urine), hematological & biochemical investigations	Screening, 90
X-ray, KOOS questionnaire score, FACIT-F score, and ECG	Baseline, 90
Need for Analgesics (Rescue Medication)	30, 60, 90, 105, 120
Safety (SAEs & AEs)	Randomization to 90
Treatment Compliance	30, 60, 90

The secondary efficacy assessment was to evaluate changes in biomarkers such as PIIANP, Cartilage Oligomeric Matrix Protein [23], Human Procollagen 2 C-terminal Peptide, C-Telopeptide of type II collagen (CTX-II), TNF-α, IL-7, IL-1, IL-6, hsCRP, MMPs (MMP-3) at screening and day 90. The changes in analgesic requirement as a rescue medication were assessed at following screening days 30th, 60th, 90th, 105th, and 120th. Changes in gastrointestinal symptoms like heartburn, gastric discomfort, and epigastric pain on the 4-point Linkert scale were assessed at following screening, days 7th, 15th, 30th, 45th, 60th, and 90th. Changes in KOOS scoring for quality of life and in FACIT-F score were assessed on day 0 and day 90. Changes in symptom grading on a 4-point Linkert scale for joint swelling, tenderness, and warmth were assessed at screening, 1st, 3rd, 7th, 15th, 30th, 45th, 60th, and 90th day (Table II).

### Safety Assessment

The safety of the investigational product was assessed by evaluating adverse events profile from baseline to the end of the study. The changes in hematological and biochemical parameters were calculated at the screening and end of the study. In addition, the investigator assessed changes in ECG at both baseline and at the end of the study for trial participants, based on their discretion. Furthermore, the compliance with the investigational product from baseline to end of the study was evaluated based on clinical examination.

### Statistical Analysis

#### Sample Size Calculation

The primary objective of this study was to assess the mean total WOMAC score between the nutraceutical and placebo groups. Based on the sample size calculation (ClinicalCalc, 2023), the study was powered at 95% with a significance level of 5% to detect a minimum clinically meaningful difference (delta) of a 30% reduction in the total WOMAC score between groups (24.6 *vs.* 17.22). The numbers provided in brackets represent the presumed mean total WOMAC scores for the two groups being compared: the nutraceutical group (24.6) and the placebo group (17.22). To achieve this, 60 completed cases were required, with an equal allocation of 30 participants to the nutraceutical and placebo groups. To account for potential dropouts, a total of 62 participants were enrolled in the study.

#### Per Protocol Subset (PP)

Participants who adhered to the study protocol without any significant violations were included in the per-protocol population. This group consisted of individuals who attended all scheduled visits and refrained from using any prohibited medications throughout the study. The case report forms (CRFs) were completed as required.

#### Modified Intention to Treat Subset (mITT)

The term “mITT” contains all randomized participants who met all inclusion/exclusion criteria, at least completed one visit and administered minimum one dose of assigned product were included in the mITT population.

#### Safety Analysis Subset

The safety population included all participants in the trial who had received at least one dosage of study products, also known as the mITT population.

#### Methods of Analysis

As per mITT, primary efficacy and secondary endpoints were evaluated using the PP population and safety variables.

#### Demographic and Baseline Information

The analysis was performed using a Student's t-test where the continuous variable, age, was comprehensively summarized using descriptive statistics, including the number of observations, mean, and standard deviation, with a 95% confidence interval assuming a normal distribution. Gender was assessed separately using the Chi-square test.

#### Analysis of Efficacy Parameters

The normality of the data was assessed using the Kolmogorov–Smirnov test. If the data were found to be non-parametric, the chi-square test was used for categorical variables. In this study, weight, height, and BMI were represented in mean ± SD. The total VAS pain score, distance covered in the 6-min walk test, WOMAC score, serum and urine biomarker levels, and FACIT-fatigue score data were analyzed using a student t-test. Between-group changes in the OA-related symptoms (joint swelling, tenderness, and warmth), as well as gastrointestinal symptoms (gastric discomfort, epigastric pain, and heartburn) data, were analyzed by Chi-square test. Decrease in reliance on analgesics is documented in the cumulative number and percentage of participants (Table II).

#### Safety Analysis

Laboratory investigations such as hematological and biochemical parameters between group data are exhibited as mean and standard deviation. The compliance was expressed in percentage. SPSS software was used to conduct all statistical analysis.

## Results

### Demographic Characteristics and Anthropometric Data of Patients

The demographic and anthropometric data of the study population comprised 60 participants. Table III presents the demographic characteristics, including gender distribution and age; no statistically significant differences were found between the groups. However, there were comparatively higher numbers of females than males in the study across groups. The effect of the treatment on the anthropometric markers is also shown on day 90. There was no statistically significant difference in the anthropometric markers between groups. In addition, the assessment of alcohol consumption and smoking/tobacco use revealed varying numbers of participants across different groups. Specifically, the nutraceutical group had the highest number of participants consuming alcohol (*n* = 8) and smoking/using tobacco (*n* = 9). The placebo group had only 3 participants consuming alcohol and 7 smoking/using tobacco. Furthermore, it is pertinent to highlight that all participants refrained from smoking and alcohol throughout the study period. No significant differences were observed in the number of participants adhering to vegetarian and non-vegetarian diets between groups.

**Table III:** Demographic Characteristics of Patients

Demographic Details	Nutraceutical (*n* = 30)		Placebo (*n* = 30)	
Male	13		10	
Age (Mean ± SD)	52.62 ± 8.15		54.6 ± 7.60	
Female	17		20	
Age (Mean ± SD)	52.29 ± 6.90		55.1 ± 6.73	
Comorbid conditions				
Hypertension	2		8	
Diabetes mellitus	3		3	
Hypercholesterolemia	1		0	
Hypothyroidism	2		2	
Baseline				
Anthropometry (mean ± SD)	Male (*n* = 13)	Female (*n* = 17)	Male (*n* = 10)	Female (*n*−20)
Weight (Kg)	59.9 ± 9.66	57.78 ± 8.28	65.33 ± 12.10	60.49 ± 10.17
Height	158.67 ± 8.00	153.02 ± 5.56	161.07 ± 7.94	155.07 ± 12.28
BMI (kg/m^2^)	23.61 ± 4.03	24.70 ± 3.34	24.99 ± 3.25	25.32 ± 4.02
Day 90				
Weight (kg)	59.42 ± 9.65	57.32 ± 8.25	64.72 ± 11.90	59.96 ± 10.16
BMI (kg/m^3^)	23.74 ± 4.61	24.50 ± 3.33	24.77 ± 3.21	25.11 ± 4.09

### Clinical Efficacy

#### Effect on Symptoms and the Physical Inability

**Fig. 2:**
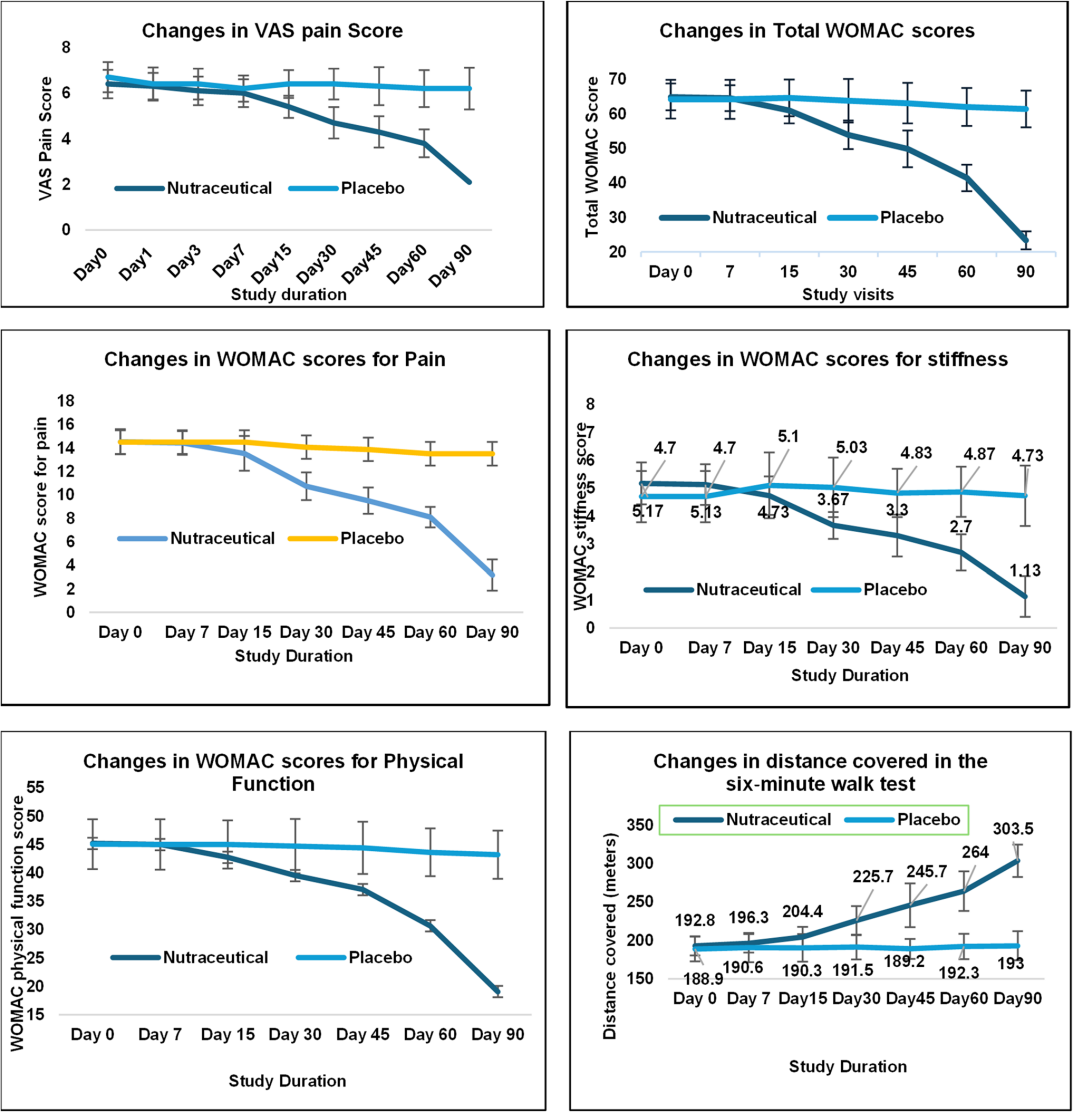
Reduction in symptoms and improved physical function by the nutraceutical by 90 days of treatment.

**Fig. 3:**
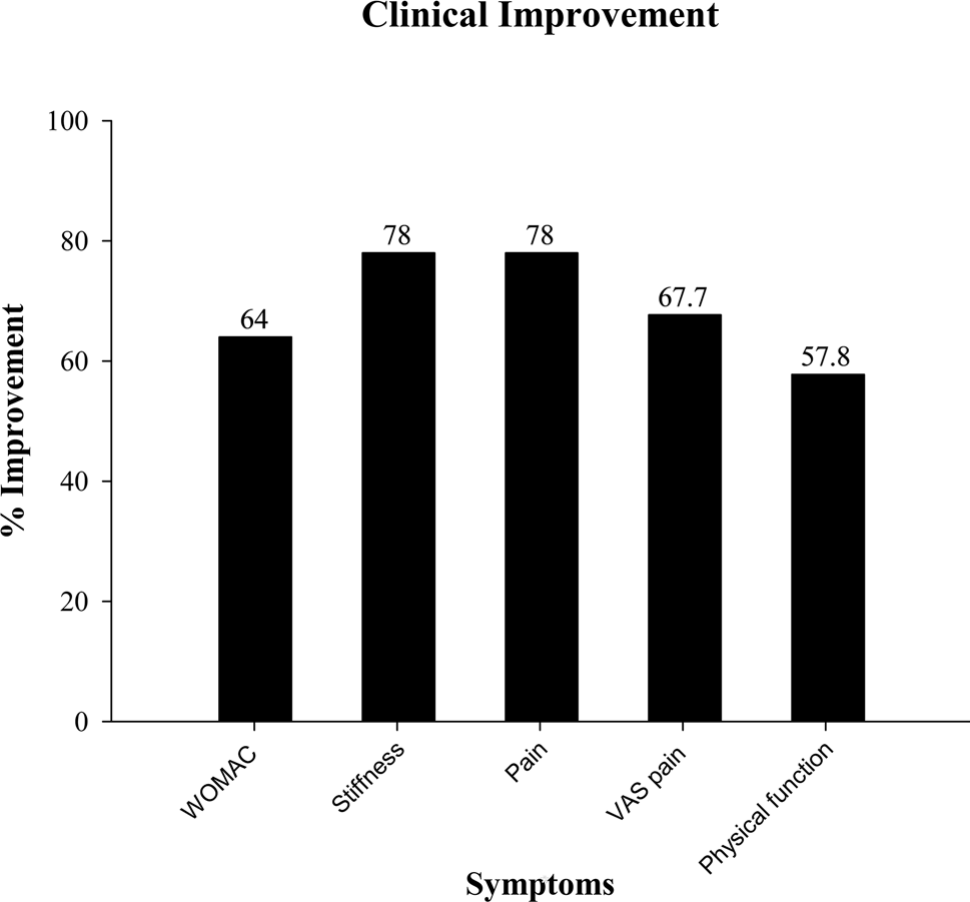
Effect of the nutraceutical on various symptoms due to osteoarthritis compared to placebo.

At the baseline, patients had mild to moderate pain with or without stiffness and tenderness in the joint. Some patients reported warmth at the affected joint and inability to do daily movements. The severity of these symptoms was examined at the baseline and the end of treatment (90 days) by VAS score of the overall pain and by WOMAC detailed questionnaire for the disability, severity of pain and stiffness (ability to do daily physical activity). Table IV gives the number of patients showing these symptoms at the baseline. As shown in Fig. 2, the VAS score at the baseline was 6.7 ± 0.66 in the placebo group and 6.4 ± 0.62 in the nutraceutical group, which reduced to 6.2 ± 0.91 (7.5%) and 2.1 ± 0.87 (67.7%) respectively. Furthermore, detailed assessment of pain and associated symptoms by the WOMAC questionnaire showed significant improvement in various symptoms. A total WOMAC score reduced from 64.90 ± 3.88 to 23.37 ± 2.58 (64%) in the treatment group (p < 0.001) compared to only a 4.4% reduction in the placebo group (64.27 ± 5.61 to 61.43 ± 5.3 p < 0.1). 78.2% reduction in WOMAC pain score was observed in the treatment group (14.53 ± 1.07 to 3.17 ± 1.32 p < 0.001) compared to the placebo group (14.50 ± 1.20 to 13.50 ± 1.41- 6.9% p < 0.01). Similarly, WOMAC stiffness score was reduced by 78% in the treatment group (5.17 ± 0.75 to 1.13 ± 0.73 p < 0.001) *vs* only 0.7% in the placebo group (4.70 ± 0.92 to 4.73 ± 1.08 p < 0.9) (Fig. 2). This symptomatic improvement is reflected in the improvement in the physical function of these patients. At the baseline, all the patients showed immobility at the score of 45.20 ± 3.21 in the treatment group and 45.07 ± 4.39 in the placebo group. At the end of the treatment, the nutraceutical-treated group showed a 57.8% reduction in the immobility score (19.07 ± 2.35 p < 0.001) compared to only 4.1% in the placebo group (43.20 ± 4.25). Patients treated with the nutraceutical also reported improvement in their walking ability. This was showed in the 6 min walk test. Both the groups showed walking inability with distance covered 192.8 ± 12.47 m (Treatment group) and 188.9 ± 16.22 m (placebo group) in 6 min at the baseline. Towards the end of 90 days of treatment nutraceutical group could cover 303.5 ± 20.99 m distance (Δ57.46 m) *vs* placebo group could walk 193.0 ± 18.80 m distance (Δ2.13 m). Figure 3 gives detailed data on symptomatic relief in the nutraceutical group. Moreover, as per the Physician's Global Assessment in the nutraceutical group, signs of improvement emerged as early as day 7, with 13.33% (*n* = 4) of participants displaying progress, escalating to 80% (*n* = 24) achieving significant improvement (much improved) by day 90, while 20% (*n* = 6) exhibited moderate improvement (improved). Conversely, the placebo group exhibited minimal change, with no significant improvement observed throughout the study. Moreover, 13.33% (*n* = 4) of participants experienced worsened symptoms at day 90, while only one participant demonstrated improved symptoms after 90 days of treatment.

**Table IV:** Number of Patients Showing Various Symptoms at the Baseline and the End of 90 Days of Treatment

Group	Score	Number of patients (%)					
		Joint swelling		Tenderness		Warmth	
		Screening	Day 90	Screening	Day 91	Screening	Day 92
Nutraceutical	0 = None	0 (0.00)	30 (100.00)	0 (0.00)	30 (100.00)	0 (0.00)	30 (100.00)
	1 = Slight	19 (63.33)	0 (0.00)	10 (33.33)	0 (0.00)	13 (43.33)	0 (0.00)
	2 = Moderate	11 (36.67)	0 (0.00)	20 (66.67)	0 (0.00)	17 (56.67)	0 (0.00)
	3 = Very	0 (0.00)	0 (0.00)	0 (0.00)	0 (0.00)	0 (0.00)	0 (0.00)
	4 = Extremely	0 (0.00)	0 (0.00)	0 (0.00)	0 (0.00)	0 (0.00)	0 (0.00)
Placebo	0 = None	0 (0.00)	0 (0.00)	0 (0.00)	0 (0.00)	0 (0.00)	0 (0.00)
	1 = Slight	11 (36.67)	19 (63.33)	7 (23.33)	16 (53.33)	13 (43.33)	23 (76.67)
	2 = Moderate	15 (50.00)	10 (33.33)	15 (50.00)	14 (46.67)	17 (56.67)	7 (23.33)
	3 = Very	4 (13.33)	1 (3.33)	8 (26.67)	0 (0.00)	0 (0.00)	0 (0.00)
	4 = Extremely	0 (0.00)	0 (0.00)	0 (0.00)	0 (0.00)	0 (0.00)	0 (0.00)

#### Radiological Findings

**Fig. 4:**
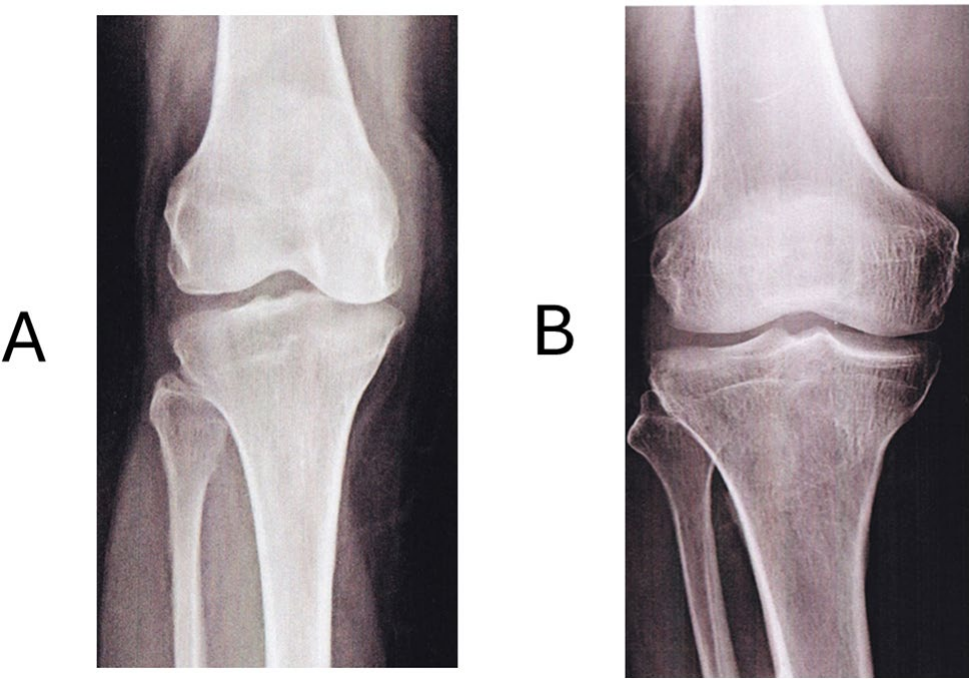
Representative radiological X-ray images of Participant #0104's Knee Before (**A**) and After (**B**) nutraceutical treatment.

All the patients underwent radiological examination (X-ray) at the baseline and end of the study. It was noted that participants from the treatment group exhibited improvement, as evidenced by increased space between the knee joints (Fig. 4). This observation aligns with the clinical findings, viz., symptomatic improvement and improvement in physical ability, reflecting in the enhanced quality of life reported by the participants & assessed by investigators.

#### Effect on Fatigue

**Fig. 5:**
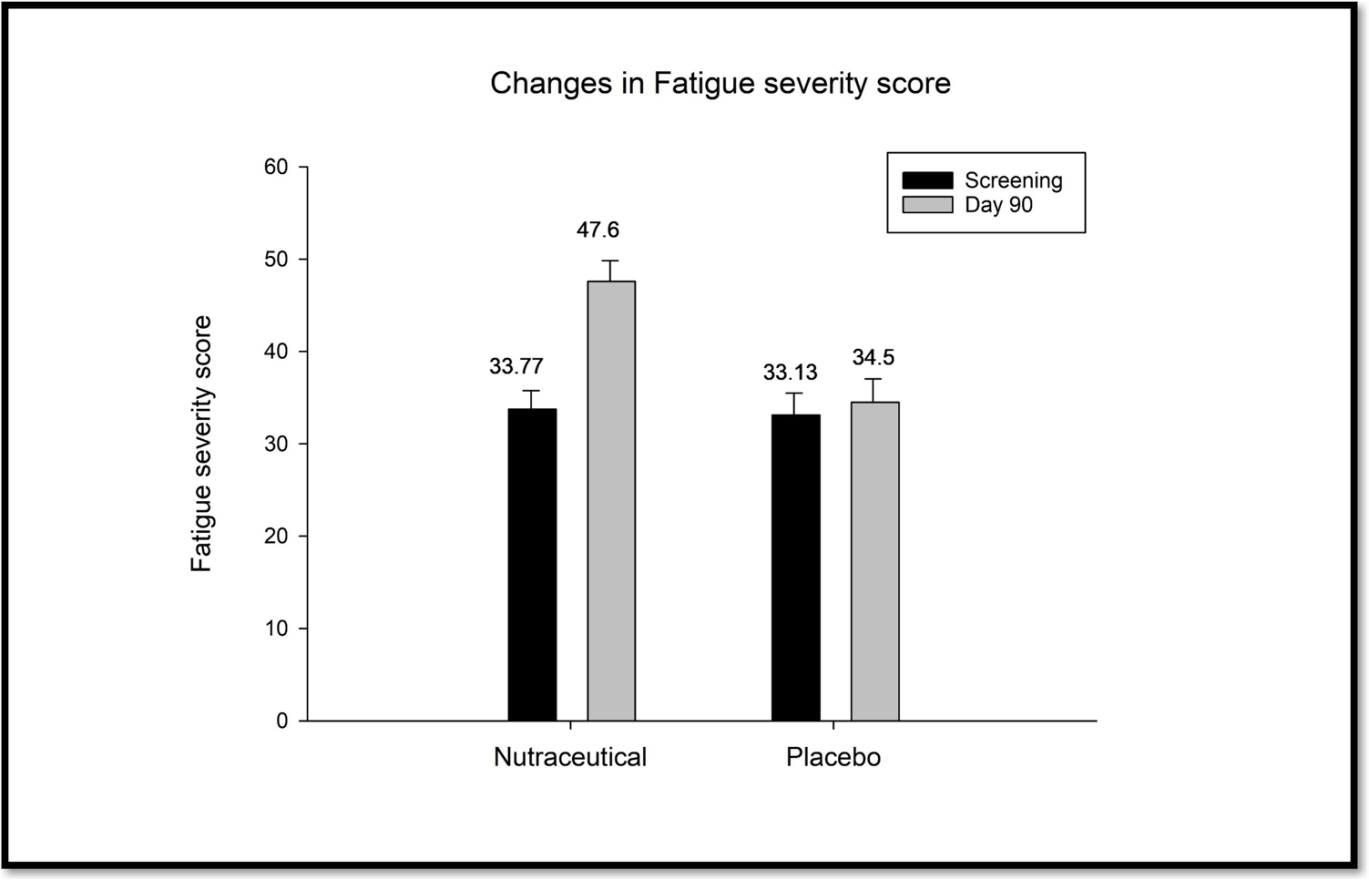
Assessment of changes in fatigue severity score between groups.

FACIT-Fatigue scale, a 13-item participant-reported measure of fatigue, scored on a response scale ranging from 0 to 4. For questions, the scoring is as follows: 0 = Not at all, 1 = A little bit, 2 = Somewhat, 3 = Quite a bit, 4 = Very much. The resulting score ranges from 0 to 52. In addition, the interpretation of the total fatigue score is as follows: A total score ≤ 30 indicates severe fatigue, 31–41 indicates moderate fatigue and 42–52 indicates mild to no fatigue. Both groups exhibited comparable levels of fatigue severity at the screening stage, with scores representing moderate fatigue, suggesting similar baseline conditions. Furthermore, after 90 days of treatment, notable differences in fatigue severity became apparent among the groups. The nutraceutical group showed the most substantial improvement, with a mean score of 47.60, indicating a 40.97% reduction in fatigue severity compared to baseline. However, the placebo group showed minimal improvement, with a mean score of 34.50, representing a 4.12% reduction in fatigue severity (Fig. 5).

#### Quality of life – KOOS Assessment

The Knee Injury and Osteoarthritis Outcome Score is a tool designed to evaluate both immediate and long-term outcomes related to knee pain and injury from the participant's viewpoint. The KOOS fulfills the key criteria for outcome measures and is effective for assessing the progression of knee pain and the results of treatment. Awareness of knee problems (Q1): participants in both groups reported significantly higher awareness levels throughout the study without significant differences between groups. Lifestyle modifications (Q2): Participants in all groups also showed a significantly higher tendency for lifestyle modifications, although the between-group difference was not significant. In the outcome of questions 3 and 4 of KOOS, the treatment group showed a substantial increase in confidence levels and reduced difficulty with the affected knee. However, the placebo group did not have such an outcome of the QOL KOOS score assessment (Table V).

**Table V:** Effect of the Nutraceutical on Quality of Life (KOOS Score)

Group	Baseline	Day 90	*P* Value	Group	Baseline	Day 90	*P* Value
**Q1. How often are you aware of your knee problems**				**Q3. How troubled are you with a lack of confidence in your knee**			
Nutraceutical (Mean ± SD)	60.83 ± 15.65	70 ± 12.11	0.003	Nutraceutical (Mean ± SD)	56.67 ± 17.29	9.17 ± 12.25	< 0.001
Placebo (Mean ± SD)	61.67 ± 14.28	67.50 ± 11.65	0.006	Placebo (Mean ± SD)	61.67 ± 12.69	61.67 ± 14.28	1
**Q2. Have you modified your lifestyle to avoid potentially damaging activities to your knee**				**Q4. In general, how much difficulty do you have with your knee**			
Nutraceutical (Mean ± SD)	24.17 ± 10.35	65.83 ± 13.90	< 0.001	Nutraceutical (Mean ± SD)	47.50 ± 12.02	13.33 ± 12.69	< 0.001
Placebo (Mean ± SD)	21.67 ± 10.85	61.67 ± 17.04	< 0.001	Placebo (Mean ± SD)	42.50 ± 14.90	44.17 ± 15.65	0.161

The data was analyzed using a dependent Student's t-test for within-group analyses. Significant at *p*-value < 0.05

#### Post-Treatment Effect: Pain and Analgesic Usage

The post-treatment effect of the investigational products was evaluated in participants over 30 days following a 90-day treatment phase. Two telephonic assessments were conducted on day 105 and day 120 to assess pain levels and the use of analgesic medications. Results indicated notable sustained improvements in pain management among patients receiving the IPs compared to those on placebo. The effect of nutraceutical on pain showed sustained relief when the VAS score was assessed after a month of treatment discontinuation. Patients did not report a recurrence of pain after discontinuing treatment, except three patients were required to take rescue medicine during the month. The VAS score in these patients remained the same—2.23 ± 0.90 and 2.30 ± 0.99 on 105 and 120, respectively, as was on day 90 (2.1 ± 0.87). In the case of a placebo group, as expected, patients continued to have similar pain as on day 90, and 23 patients were required to take the rescue medicine. The data has been provided in Table VI. Overall, these findings indicate a long-term effect of the nutraceutical capsules in managing pain, even after the completion of the treatment period. The sustained pain relief observed in the nutraceutical group highlights the potential long-term benefits of this intervention.

**Table VI:** Post-Treatment Effect of the Nutraceutical

Post-treatment				
VAS score (Mean ± SD)	Groups	**By Day 90**	**Day 105**	**Day 120**
	Nutraceutical	2.1 ± 0.87	2.23 ± 0.90	2.30 ± 0.99
	Placebo	6.2 ± 0.91	6.40 ± 0.72	6.30 ± 0.75
Analgesic requirement	Nutraceutical	3	0	1
	Placebo	23	8	13

### Effect on the Disease Pathogenesis: Inflammation

**Fig. 6:**
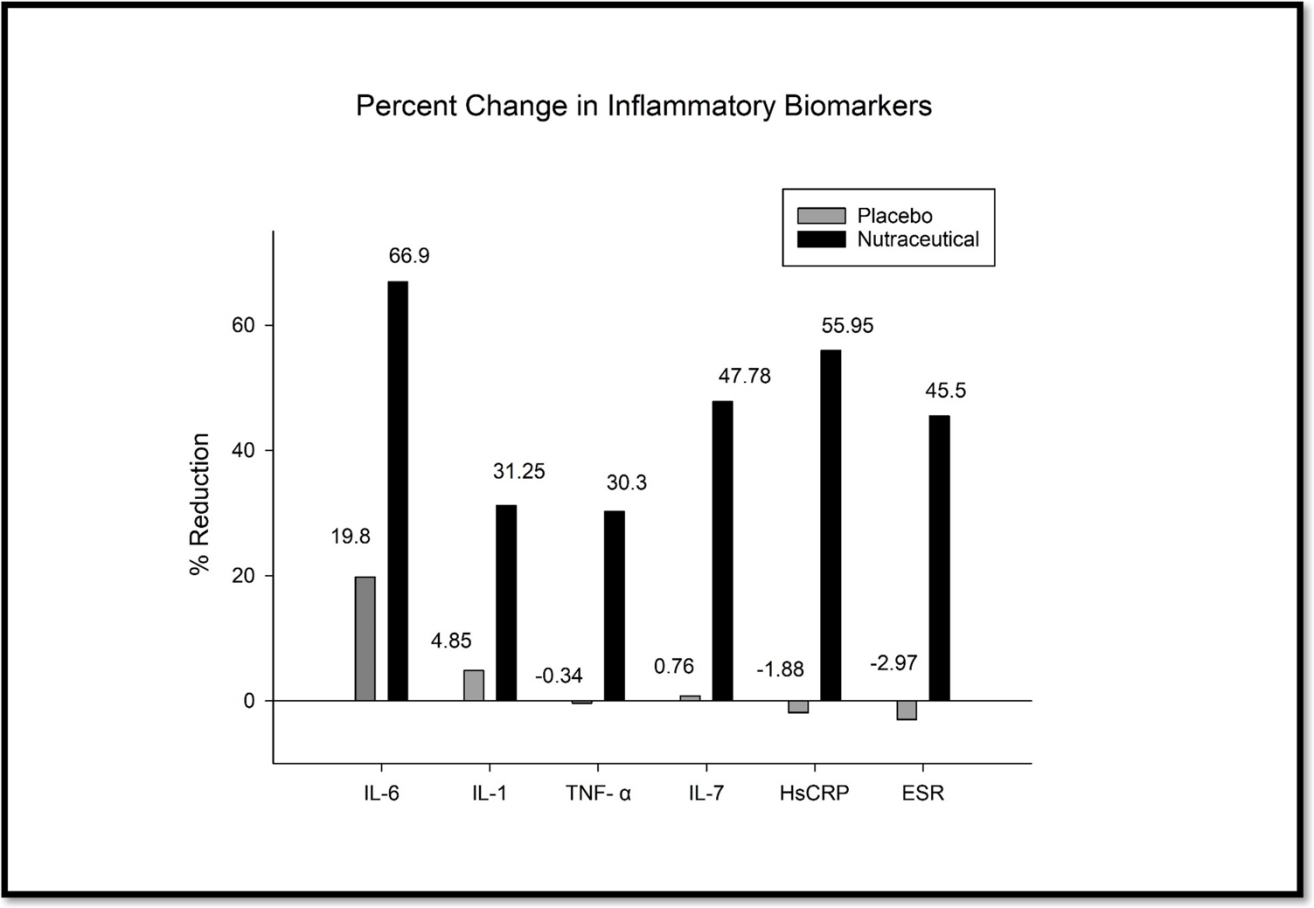
Effect of the nutraceutical on inflammatory markers.

Inflammation is a key factor contributing to the varied symptoms of osteoarthritis, viz. pain, stiffness, swelling and warmth. Prolonged inflammation further destroys cartilage in OA [24, 25]. Hence, the inflammatory status of patients was determined during the study. Inflammatory cytokines—TNF-α, IL-7, IL-1, IL-6, and other inflammatory markers hsCRP and ESR were monitored (Fig. 6 and Table VII).

**Table VII:** Effect of the Nutraceutical on Inflammatory Markers

Marker	Time	Nutraceutical	*p* value	Placebo	*p* value
IL-6 pg/ml (Mean ± SD)	Baseline	9.58 ± 4.14	< 0.001	9.84 ± 3.17	0.07
	Day 90	3.19 ± 1.48		7.85 ± 3.12	
IL-6 (% decrease from baseline)		66.69		20.19	
IL-1 pg/ml (Mean ± SD)	Baseline	10.82 ± 3.92	0.001	11.57 ± 3.30	0.062
	Day 90	7.44 ± 3.39		11.01 ± 2.99	
IL-1 (% decrease from baseline)		31.25		4.85	
TNF- α pg/ml (Mean ± SD)	Baseline	3.61 ± 0.55	< 0.001	3.99 ± 0.74	0.5
	Day 90	2.52 ± 1.09		4.00 ± 1.19	
TNF-α (% decrease from baseline)		30.3		−0.34	
IL-7 pg/ml (Mean ± SD)	Baseline	7.55 ± 2.45	< 0.001	7.85 ± 4.83	0.9
	Day 90	3.94 ± 1.57		7.79 ± 3.48	
IL-7 (% decrease from baseline)		47.78		0.76	
HsCRP µg/ml (Mean ± SD)	Baseline	5.05 ± 0.68	< 0.001	5.48 ± 1.101	0.8
	Day 90	2.22 ± 1.07		5.58 ± 2.49	
HsCRP (% decrease from baseline)		55.95		−1.88	
ESR mm/hr (Mean ± SD)	Baseline	24.7 ± 19.8	0.004	22.5 ± 14.8	0.8
	Day 90	13.5 ± 4.2		23.1 ± 14.5	
ESR (% decrease from baseline)		45.55		−2.97	

The data was analyzed using paired student t-tests. Significant at *p*-value < 0.05

#### Interleukin 6 (IL-6)

Serum IL-6 levels were comparable in both groups at the baseline. The nutraceutical group achieved a substantially more significant reduction (66.69%) in IL-6 levels than the placebo group (20.19%). The mean IL-6 level at screening was 9.58 pg/ml in the nutraceutical group, which was reduced to 3.19 pg/ml by day 90 (p < 0.001). On the contrary, the placebo group showed marginal reduction, with the mean level decreasing from 9.84 pg/mL at screening to 7.85 pg/mL at Day 90 (*p* = 0.07).

#### Interleukin 1 (IL-1)

During a 90-day study, pro-inflammatory cytokine IL-1 levels were analyzed. Initially, IL-1 levels were similar in the nutraceutical and placebo groups. After 90 days, the nutraceutical group showed a significant 31.25% reduction in IL-1 levels (10.82 ± 3.92 to 7.44 ± 3.39 pg/ml *p* < *0.001*), while the placebo group experienced a modest, and slight decrease of 4.85%(11.57 ± 3.30 to 11.01 ± 2.99 pg/ml *p* = *0.069*).

#### Tumor Necrosis Factor α (TNF-α)

Serum TNF-α levels were measured at the baseline and on Day 90 of the treatment. There was a significant reduction of 30.3% in the nutraceutical group after 90 days of treatment. In contrast, the placebo group exhibited minimal changes in TNF levels, with a non-significant 0.34% decrease. At baseline, the mean TNF-α levels in the nutraceutical group were comparable to the placebo group. However, by Day 90, the nutraceutical group demonstrated a significant decrease (3.61 ± 0.55 to 2.52 ± 1.09 pg/ml, *p* < *0.001*) in the mean TNF-α levels, while the placebo group exhibited a negligible drop (3.99 ± 0.74 to 4.00 ± 1.19pg/ml *p* = 0.5).

#### Interleukin 7 (IL-7)

At baseline, the mean IL-7 level in the nutraceutical group was comparable to the placebo group. However, by Day 90, the nutraceutical group demonstrated a significant 47.78% decrease (7.55 ± 2.45 to 3.94 ± 1.57 pg/ml, *p* < 0.001) in mean IL-7 levels, while the placebo group exhibited only 0.76% non significant decrease (7.85 ± 4.83 to 7.79 ± 3.48 pg/ml, *p* = 0.9).

#### High-Sensitive C-Reactive Protein (HsCRP)

At baseline, both groups had comparable mean hs-CRP levels (nutraceutical: 5.05 µg/ml, placebo: 5.48 µg/ml). However, by Day 90, the nutraceutical group demonstrated a substantial 55.95% reduction in mean hs-CRP levels, significantly decreasing to 2.22 µg/ml (*p* < *0.001*). In contrast, the placebo group exhibited a marginal but statistically insignificant increase in mean hs-CRP levels by 1.88 (*p* = *0.8*). Substantially reduced hsCRP levels were observed in the nutraceutical-treated group compared to the placebo.

#### Erythrocyte Sedimentation Rate (ESR)

The Assessment ESR revealed a significant reduction in the nutraceutical group at the end of the study. The mean ESR level in the nutraceutical group at screening was 24.7 mm/hr. By day 90, a substantial 45.55% decrease was observed, with the mean ESR level significantly decreasing to 13.5 mm/hr (p < 0.005). In contrast, the placebo group exhibited minimal changes in ESR levels, with a non-significant 2.97% increase (22.5 ± 14.8 to 23.1 ± 14.5 *p* = *0.8*).

### Effect on the Disease Pathogenesis: Bone and Cartilage

**Fig. 7:**
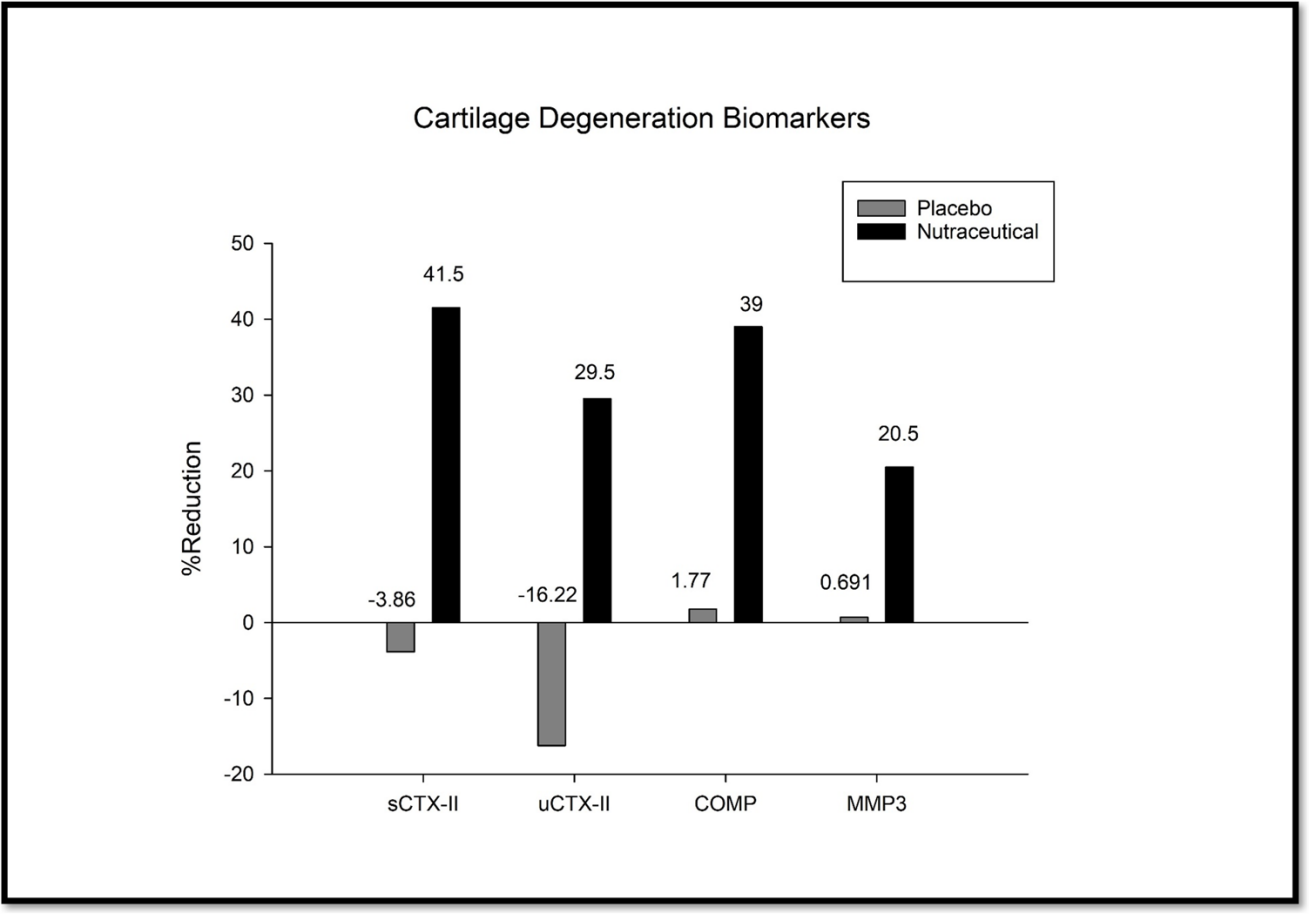
Effect of the nutraceutical on the degeneration of cartilage.

Key biomarkers such as increased CTX-II, COMP and MMP3 and reduced serum PIICP and PIIANP have been identified as potential indicators of radiographic OA progression, reflecting early joint destruction and articular damage in affected individuals [26]. The effect of the nutraceutical on these markers was investigated during the study (Fig. 7).

#### Serum and Urine C-Terminal Cross-Linked Telopeptides of Type II Collagen (CTX-II)

Serum levels of CTX-II were comparable between the nutraceutical group (10.03 ± 1.71 ng/mL) and the placebo group (10.28 ± 1.87 ng/mL) at the baseline. However, after the 90-day treatment period, the nutraceutical group exhibited a significant 41.40% reduction to 5.88 ± 1.00 ng/ml. As compared to the treated group, the placebo group showed a modest (3.86%) increase to 10.67 ± 2.68 ng/ml, which was not statistically significant. At the baseline, urine CTX-II levels were comparable between the nutraceutical group (1.68 ± 0.51 ng/ml) and the placebo group (1.61 ± 0.28 ng/ml). After the 90-day treatment period, the nutraceutical group demonstrated a 29.45% reduction in urine CTX-II levels (1.18 ± 0.23 ng/ml). Conversely, the placebo group exhibited a 16.22% increase in urine CTX-II (1.92 ± 0.30 ng/ml) over the same period.

#### Cartilage Oligomeric Matrix Protein (COMP)

At baseline, COMP levels were comparable between the groups. After 90 days of treatment, the nutraceutical group showed a significant 38.9% (from 19.02 ± 3.49 µg/ml to 11.60 ± 3.77 µg/ml, *p* < *0.001*) reduction in COMP levels. In contrast, the placebo group exhibited a non-significant 3.58% decrease (from 18.37 ± 3.34 µg/ml to 17.71 ± 3.55 µg/ml) in COMP levels. Comparing both groups after 90 days, there was a substantial difference in outcomes with enhanced efficacy for the nutraceutical.

#### Matrix Metalloprotein (MMP-3)

At the baseline, MMP-3 levels were comparable between the groups. After 90 days of treatment, the nutraceutical group showed a significant 46.43% reduction (from 38.28 ± 9.23 ng/ml to 20.51 ± 6.79 ng/ml *p* < *0.001*) in MMP-3 levels. In contrast, the placebo group exhibited a non-significant 4.17% decrease in MMP-3 levels (from 37.36 ± 9.81 ng/ml to 35.80 ± 8.44 ng/ml). Comparing both groups after 90 days, there was a considerable difference in outcomes with enhanced efficacy for the nutraceutical.

#### Procollagen 2 N-Terminal Peptide (PIIANP)

**Fig. 8:**
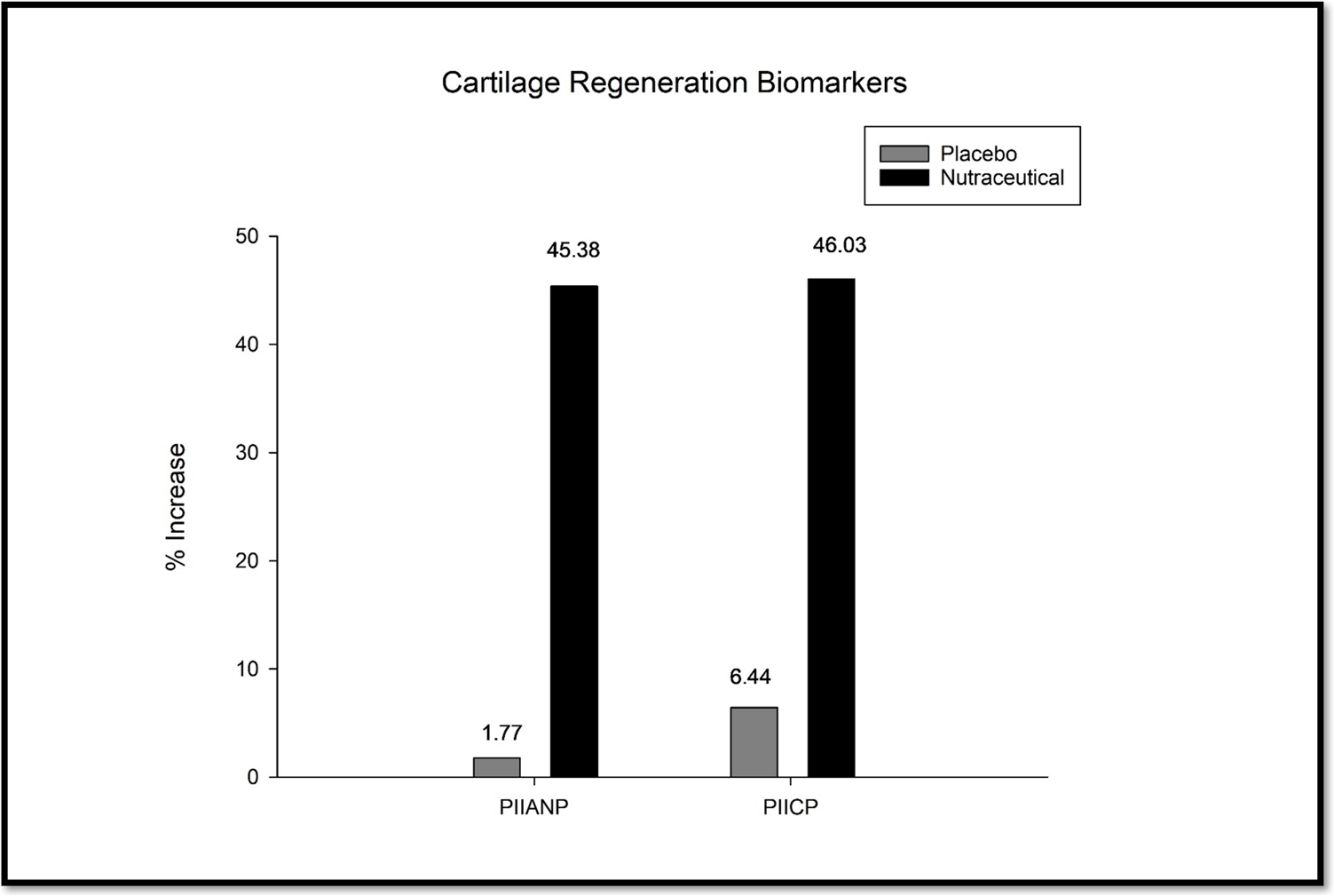
Effect of the nutraceutical on the regeneration of cartilage.

The procollagen II N-propeptide (PIIANP) levels are biomarkers of type II collagen synthesis and cartilage formation. At the baseline, PIIANP levels were comparable between the nutraceutical group (11.46 ng/ml) and the placebo group (11.37 ng/ml). After the 90-day treatment period, the nutraceutical group displayed a substantial 45.38% increase in PIIANP levels (16.67 ng/m, *p* < *0.001*) compared to the baseline value (Fig. 8). In contrast, the placebo group exhibited only a modest 1.77% increase (11.57 ng/ml), which was insignificant. The marked elevation in PIIANP levels observed in the nutraceutical group suggests a potential anabolic effect on cartilage matrix formation and repair conferred by the treatment.

#### Procollagen 2 C-Terminal Peptide (PIICP)

The serum levels of PIICP were comparable between groups at screening. As shown in Fig. 8, the nutraceutical group exhibited an increase in PIICP levels by 46.03%. However, the reduction in the placebo group was relatively small, at only 6.44%. The nutraceutical group showed PIICP levels of 443.14 ± 74.15 ng/ml at the baseline, which increased to 647.13 ± 73.47 ng/ml (*p* < *0.001*). Comparing both groups after 90 days, there was a substantial difference in outcomes, with enhanced efficacy for the nutraceutical (Fig. 8 and Table VIII).

**Table VIII:** Effect of the Nutraceutical on the Markers of Disease Pathogenesis

Marker	Time	Nutraceutical	*p* value	Placebo	*p* value
Serum CTX-2 (ng/mL) (Mean ± SD)	Baseline	10.03 ± 1.71	< 0.001	10.28 ± 1.87	0.35
	Day 90	5.88 ± 1.00		10.67 ± 2.68	
Serum CTX-2 (% decrease from baseline)		41.4		3.77	
Urine CTX-2 (ng/mL) (Mean ± SD)	Baseline	1.68 ± 0.51	< 0.001	1.61 ± 0.28	0.005
	Day 90	1.18 ± 0.23		1.92 ± 0.30	
Urine CTX-2 (% decrease from baseline)		29.45		−16.22	
COMP (ug/mL) (Mean ± SD)	Baseline	19.02 ± 3.49	< 0.001	18.37 ± 3.34	0.09
	Day 90	11.60 ± 3.77		17.71 ± 3.55	
COMP (% decrease from baseline)		38.99		3.58	
MMP-3 (ng/mL) (Mean ± SD)	Baseline	38.28 ± 9.23	< 0.001	37.36 ± 9.81	0.19
	Day 90	20.51 ± 6.79		35.80 ± 8.44	
MMP-3 (% decrease from baseline)		46.43		4.17	
PIIANP (ng/mL) (Mean ± SD)	Baseline	11.46 ± 3.45	< 0.001	11.37 ± 2.04	1.77
	Day 90	16.67 ± 4.05		11.57 ± 2.33	
PIIANP (% decrease from baseline)		45.38		1.77	
PIICP (ng/mL) (Mean ± SD)	Baseline	443.14 ± 74.15	< 0.001	452.50 ± 67.75	0.255
	Day 90	647.13 ± 73.47		481.65 ± 139.54	
PIICP (% decrease from baseline)		46.03		6.44	

The data was analyzed using paired student t-tests. Significant at *p*-value < 0.05

### Safety Assessment

The safety of the investigational treatment was monitored clinically throughout the study. Patients were carefully monitored for adverse events through clinical signs and symptoms during examinations, or if any participant reported issues. In addition, effects on hematological and biochemical markers were monitored to ensure hematological and organ safety. No severe events were observed throughout the study and after a month of treatment discontinuation. Patient compliance was 100% with the investigational product and was not affected by any adverse effects. No clinically significant changes were noted in hematological and biochemical investigations, indicating the safety of the intervention (Table IX).

**Table IX:** Assessment of Hematological and Biochemical Investigations

Blood Parameters	Nutraceutical		Placebo	
Complete Blood Count (Mean ± SD)				
	Day 0	Day 90	Day 0	Day 90
Total Leukocyte Count/µl	6903.3 ± 1705.1	6891.1 ± 1490.6	8196.7 ± 2079.2	7300.6 ± 1563.3
Neutrophils (%)	55.9 ± 7.7	56.4 ± 7.0	56.9 ± 8.5	57.7 ± 8.6
Lymphocytes (%)	34.9 ± 7.4	34.1 ± 6.9	34.0 ± 8.1	33.2 ± 8.7
Monocytes (%)	5.7 ± 1.1	5.9 ± 0.9	5.7 ± 1.5	5.7 ± 1.4
Eosinophil (%)	3.5 ± 0.6	3.6 ± 0.7	3.2 ± 1.0	3.4 ± 1.1
Basophils (%)	0.0 ± 0.0	0.0 ± 0.0	0.0 ± 0.0	0.0 ± 0.0
Total RBC Count × 10^6^/ µl	4.5 ± 0.5	4.7 ± 0.6	4.5 ± 0.6	4.7 ± 0.6
Platelet count × 103/ µl	258.3 ± 78.7	284.8 ± 90.2	293.7 ± 72.3	293.1 ± 78.5
Hemoglobin %	12.9 ± 1.4	12.9 ± 1.1	12.5 ± 1.1	13.6 ± 1.1
Hematocrit %	38.6 ± 5.3	40.1 ± 5.8	39.1 ± 3.9	42.8 ± 6.1
ESR mm/hr	24.7 ± 19.8	13.5 ± 4.2	22.5 ± 14.8	23.1 ± 14.5
Liver Function Test				
Total Bilirubin mg/dl	0.8 ± 0.5	0.6 ± 0.3	0.6 ± 0.2	0.6 ± 0.2
Direct Bilirubin mg/dl	0.2 ± 0.2	0.2 ± 0.1	0.2 ± 0.1	0.2 ± 0.1
Indirect Bilirubin mg/dl	0.5 ± 0.3	0.5 ± 0.2	0.4 ± 0.2	0.5 ± 0.2
SGOT/ AST U/l	25.5 ± 10.1	28.2 ± 7.0	29.1 ± 11.9	30.4 ± 7.2
SGPT/ ALT U/l	25.2 ± 9.9	23.7 ± 8.9	25.8 ± 10.4	27.7 ± 8.8
Alkaline Phosphatase U/l	156.5 ± 57.7	180.7 ± 67.8	164.0 ± 56.5	183.0 ± 56.4
Total Protein gm/dl	7.3 ± 0.7	7.5 ± 0.6	7.1 ± 0.7	7.4 ± 0.5
Albumin g/dl	4.1 ± 0.4	4.1 ± 0.4	4.1 ± 0.4	4.2 ± 0.4
Globulin gm/dl	3.2 ± 0.5	3.3 ± 0.6	3.0 ± 0.6	3.2 ± 0.5
A/G Ratio	1.3 ± 0.2	1.3 ± 0.2	1.4 ± 0.4	1.3 ± 0.2
Renal Function Test				
Blood Urea Nitrogen mg/dl	9.7 ± 2.5	9.8 ± 3.6	11.0 ± 2.6	11.4 ± 3.6
Blood Urea mg/dl	20.7 ± 5.4	20.9 ± 7.8	23.7 ± 5.6	24.3 ± 7.7
Serum Creatinine mg/dl	0.8 ± 0.2	0.8 ± 0.1	0.8 ± 0.2	0.9 ± 0.1
Uric Acid mg/dl	4.9 ± 1.5	5.0 ± 1.1	4.7 ± 1.2	5.4 ± 1.2

Additionally, sustained effects of the intervention were observed during the post-treatment period in effectively managing pain and reducing analgesic dependency in the nutraceutical group compared to placebo. Osteoarthritic patients often report gastrointestinal problems, primarily due to long-term use of analgesics (NSAIDs) and other antiarthritic drugs [27]. In the present study, patients did not report any gastrointestinal disturbances during and after a month of discontinuation. On the contrary, all the patients with mild to moderate baseline gastric issues reported significant reductions in heartburn, gastric discomfort, and epigastric pain in the nutraceutical group, with 100% of patients experiencing improvement (Table X). Additionally, the need for analgesic medication decreased significantly in the nutraceutical group, while the placebo group showed the highest cumulative percentage of participants requiring analgesics.

**Table X:** Effect of the Nutraceutical on Gastrointestinal Symptoms

Group	Score	Gastrointestinal Symptoms- Score and Number of Patients (%)					
		Gastric discomfort		Epigastric pain		Heart Burn	
		Baseline	Day 90	Baseline	Day 90	Baseline	Day 90
Nutraceutical	0 = None	9 (30)	18 (60)	9 (30)	18 (60)	13 (43.3)	17 (56.7)
	1 = Mild	16 (53.3)	12 (40)	16 (53.3)	12 (40)	11 (36)	13 (43.3)
	2 = Moderate	5 (16.7)	0	5 (16.7)	0	6	0
	3 = Severe	Nil					
Placebo	0 = None	7 (23.3)	8 (26.7)	7 (23.3)	8 (26.7)	9 (30)	9 (30)
	1 = Mild	15 (50)	15 (50)	15 (50)	15 (50)	14 (46.7)	14 (46.7)
	2 = Moderate	8 (26.7)	7 (23.3)	8 (26.7)	7 (23.3)	7 (23.3)	7 (23.3)
	3 = Severe	Nil					
	*P* value*	0.059	0.009	0.012	< 0.001	0.28	0.037

^*^ Data is represented as number of participants (percentage). Data analyzed by chi-square test, Significant at *p* < 0.05

## Discussion

The present placebo-controlled double-blind clinical study reports the effect of a nutraceutical combination of extracts from *Boswellia serrata* gum and *Apium graveolens* L. seed, in osteoarthritis. These plants have been extensively utilized in traditional medicine as natural remedies for both acute and long-term inflammatory or painful illnesses, and their extracts have been effective in various *in vitro* and *in vivo* studies [17, 28]. The effect of the nutraceutical in osteoarthritis gave prolonged symptomatic relief, reduced inflammation, and modulated the disease pathogenesis by preventing degeneration and inducing cartilage regeneration. The nutraceutical exhibited a significant reduction compared to the placebo in osteoarthritis-induced pain, stiffness, swelling, and redness, as evaluated by the WOMAC score and the VAS pain score. It improved physical function, mobility, and flexibility. Towards the end of 90 days of treatment with the nutraceutical, the total WOMAC score decreased by 64%, with a 78% decrease in stiffness and pain also 58% improvement in physical function. The VAS pain score decreased by 67%. This enhanced functional capacity, as the WOMAC immobility score was reduced by 57.8% and the patient could walk 57.4 m extra distance in the 6 min walk test compared to the baseline. This clinical improvement was reflected in increased independence, reduced fatigue by < 40%, and improved well-being and, thereby, overall quality of life as determined by the KOOS assessment. As per the physician's global Assessment, this clinical improvement was seen as early as seven days of treatment in 13.3% of patients (*n* = 4). By day 90, 80% (*n* = 24) achieved significant improvement, while 20% (*n* = 6) exhibited complete improvement. This clinical improvement was sustained even after a month of treatment discontinuation, as assessed by the VAS score. Also, in comparison with previously published interventions for osteoarthritis, the improvement in VAS Pain score in Appelboom *et al.* [29] with avocado/soybean unsaponifiables for 3 months was 55.43% and in Hashempur *et al.*[30] with green tea (*Camellia sinensis*) for 4 weeks was 25%, whereas our study showed 67.7% improvement in VAS Pain score. Patients did not report a recurrence of pain after discontinuing treatment, except for the three patients who were required to take rescue medicine during the month. The nutraceutical significantly reduced the requirement for rescue medication. Notably, over 40% improvement in total WOMAC scores was observed in 100% (*n* = 30) of participants in the nutraceutical group, while 0% of participants in the comparator group reached this threshold, all remaining below 40%. In comparison with previous published interventions for osteoarthritis, the improvement in total WOMAC score in Ghoochani *et al.* [31] with pomegranate juice for 6 weeks was 18.34%, Kuptniratsaikul *et al.* [32] with *Curcuma domestica* for 4 weeks was 36.60%, Hashempur *et al.* [30] with green tea (*Camellia sinensis*) for 4 weeks was 24.72%, Altman and Marcussen *et al.* [33] with ginger species (*Zingiber officinale* and *Alpinia galanga*) for 6 weeks was 25.70%, Pagonis *et al.* [34] *with* Methylsufonylmethane for 26 weeks was 36.94%, Jacquet *et al.* [35] with Phytalgic (fish-oil, vitamin E, *Urtica dioica)* for 4 weeks was 57.02% and Arden *et al.* [36] with Vitamin D for 3 years was 99.69%, whereas our study showed 64% improvement in total WOMAC score. In addition, the improvement in WOMAC Pain score in previously published studies for osteoarthritis was 79.38% with fish oil for 24 months [37]*,* 7.92% [31], 38.68% [32], 35.89% [30], 26.33% [33], 36.82% [34], 59.90% [35] and 100.24% [36], whereas our study showed 78% improvement in WOMAC Pain score. Furthermore, the improvement in WOMAC Stiffness score for osteoarthritis was 43.02% [31], 35.69% [32], 28.26% [30], 30.85% [33], 39.18% [34], 58.01% [35] and 104.30% [36], whereas our study showed 78% improvement in WOMAC Stiffness score. Moreover, the improvement in WOMAC Physical function for osteoarthritis was 18.78% [31], 35.66% [32], 20.71% [30], 23.84% [33], 45.07% [34], 56.21% [35], 84.26% [37] and 98.83% [36], whereas our study showed 57.8% improvement in WOMAC Physical function.

Conversely, the placebo group showed no improvement, with some patients experiencing worsened pain, highlighting the effectiveness of the study medications. Similar symptomatic relief was reported by Desai *et al*. [38] using a similar extract of Boswellia and celery in adults afflicted by chronic inflammatory conditions, including osteoarthritis and rheumatoid arthritis. Concomitantly, the nutraceutical in this study increased the knee joint gap, as seen in the radiological assessment by X-ray, which explains the symptomatic relief and the clinical improvement seen in this study. The nutraceutical also reduced inflammation in the patients, a key pathogenic mechanism which contributes to the varied symptoms of osteoarthritis following destruction of the cartilage in OA [24, 25]. The maximum effect was seen on IL-6, with a 66.9% reduction. IL-6 is central to the inflammatory cascade in OA through i) an increase in the expression of other inflammatory mediators, viz*.*, ROS, IL-1β and TNF-α, in chondrocytes and cartilage, ii) induction of the migration of neutrophils into the joints [39], iii) acting on synovial fibroblasts and chondrocytes which leads to a further rise in IL-6 in the joint. These events collectively create a chronic inflammatory milieu in the affected joint and induce cartilage degeneration and bone loss [40, 41]. The nutraceutical also reduced other inflammatory cytokines—IL1 (31%), TNF-α (30%) and IL-7 (47%). This was reflected in the 55% reduction in HsCRP, and 45% reduction in ESR levels by the 90-days of treatment. ESR and Hs- CRP also serve as markers of inflammation. Low-grade inflammation in OA is associated with mild to moderate elevation of HsCRP and ESR, contributing to the clinical features of tenderness, swelling, patellar ballottement and muscle weakness [42]. These findings demonstrate the anti-inflammatory activity of this nutraceutical combination.

Dextrin (used in our placebo capsules) has indeed been investigated for induction of changes in the gut microbiota as a prebiotic. However, these changes are seen only when dextrin is administered in dosages falling in the gram ranges, typically 10 g daily intake as evidenced by published studies in the literature on dextrin as a prebiotic [43, 44]. In our study, the dose of dextrin administered in our placebo capsules was comparatively minuscule, i.e., 200 mg per capsule. Since we administered two capsules per day, the total amount of dextrin administered was only 400 mg/day. Hence, in the dose used in this study, dextrin is highly unlikely to result in adverse events or beneficial events in inflammation.

In addition, to see if the nutraceutical affects cartilage and bone, several biomarkers were assessed in the subjects at baseline and the end of treatment. The collagen degradation product CTX-II is a prognostic marker of OA. Increased serum and urinary CTX-II levels suggest structural changes, viz., cartilage degradation, bone remodelling, and osteophyte formation in the affected joint, narrowing the joint spaces, increasing subchondral bone thickness, and synovial inflammation [45]. Nutraceutical-treated patients showed a 41.4% reduction in sCTX-II and a 29.45% reduction in uCTX-II over baseline by 90 days of treatment, which indicated prevention and/or reduction of cartilage degeneration in the treatment group. Similarly, other degeneration markers of OA – COMP and MMP3 showed a decrease in serum levels by 38% and 46%, respectively. COMP, a cartilage degeneration marker, also contributes to structural alteration through increased subchondral bone volume, spurs at the joint margins, thickening and fibrosis of ligaments, and damage to the menisci [46]. MMP-3 is involved in the degeneration of the cartilage extracellular matrix, which leads to fibrillation and erosion. It also contributes to synovial membrane thickening and inflammation, facilitates osteophyte formation at joint margins, remodelling of the subchondral bone, and induces sclerosis and cyst formation [47]. Increasing levels of COMP and MMP3 collectively suggest cartilage degeneration, and the nutraceutical prevented this degradation significantly compared to placebo. It was interesting to see that the nutraceutical prevented this degradation and increased collagen synthesis, which is a rare finding suggesting cartilage regeneration. There was a significant increase in the cartilage regeneration markers – PIIANP (45%) and PIICP (46%). A previous study on knee osteoarthritis found that an 8-week exercise program could increase synthesis of type II collagen and reduce collagen degradation. This was evidenced by a substantial increase in PIICP levels and improvement in physical performance [48]. Furthermore, regarding safety assessment, no adverse effects or untoward reactions were noted. The biochemical and hematological profiles at baseline and day 90 were similar and did not indicate any abnormal changes. On the contrary, patients reporting gastrointestinal issues at baseline showed improvement.

Obese patients were excluded from this study to minimize potential confounding factors, as obesity (BMI ≥ 30.00 kg/m^2^) is a well-established risk factor for OA progression. By including only participants with a BMI < 30.00 kg/m^2^, which encompasses normal-weight and overweight individuals, the study allowed for a more controlled assessment of the intervention's effects on OA without the additional impact of obesity. Obesity contributes to OA through increased joint loading and systemic inflammation, which could independently influence clinical outcomes. Therefore, excluding obese individuals ensured a clearer evaluation of the intervention's effectiveness, free from the complexities associated with obesity. Future trials involving obese participants are planned to assess the intervention's efficacy and safety.

Both *in vivo* and *in vitro* studies indicate that formulations containing *Boswellia serrata* may inhibit the catabolic activities of key inflammatory mediators in the early stages of OA and obstruct inflammatory pathways associated with its progression. Specifically, 3-O-Acetyl-11-keto-beta-boswellic acid (AKBA) in *Boswellia serrata* extract exerts anti-inflammatory effects by inhibiting 5-lipoxygenase, a primary source of pro-inflammatory leukotrienes [49, 50]. A recent meta-analysis (2020) demonstrated that *Boswellia serrata* extract is both safe and effective for OA patients [51]. These findings are consistent with the present study, which showed that the nutraceutical effectively alleviated symptoms associated with knee OA through its analgesic and anti-inflammatory properties. The mechanistic underpinnings of *Boswellia*'s anti-inflammatory action, speculated to involve modulation via prostaglandin E synthase-1 and the serine protease cathepsin G [52], offer additional insights into its efficacy. Clinical trials have consistently highlighted the positive impact of *Boswellia serrata* oleoresin on pain, mobility, and swelling in knee OA patients, affirming its therapeutic value [1]. Moreover, the antioxidative, analgesic, and anti-inflammatory properties of *Apium graveolens *via inhibition of TNF-α and NF-κB through its ability to inhibit proinflammatory cytokines and modulate oxidative stress pathways presents an alternative to conventional OA management strategies [53].

The study features a randomized, double-blind, multicentric, and placebo-controlled design, which enhances the credibility of the findings by minimizing bias and improving reliability. Additionally, the use of validated scales and measures reinforce the study's validity, ensuring accurate assessments of outcomes. However, to enhance the generalizability of the results, future research should focus on larger-scale trials with extended follow-up periods, particularly involving older populations and individuals with advanced or severe osteoarthritis.

## Conclusions

The combination therapy of *Boswellia serrata* (300 mg) and celery seed extract (250 mg) yielded the most pronounced benefits compared to the placebo. It gave prolonged symptomatic relief, reduced inflammation, inhibited cartilage degeneration and induced its synthesis. It was found safe over 90 days of treatment at the given doses. The remarkable efficacy of the nutraceutical combination could be attributed to the synergy achieved by combining high doses of *Boswellia serrata* and celery seed extract. Hence, this nutraceutical may serve as a promising formulation for the management of osteoarthritis. Additional research may be necessary to clarify the long-term efficacy and safety in a larger cohort.

## Supplementary Information

Below is the link to the electronic supplementary material.

Figure S1 Representation of the measured components of nutraceutical using HPLC-PDA (PNG 400 KB)

High Resolution Image (TIF 1865 KB)

## Data Availability

The datasets generated during and/or analysed during the current study are available from the corresponding author on reasonable request.
