# Supplementary figures and images for: Efficacy and Safety of Boswellia serrata and Apium graveolens L. Extract Against Knee Osteoarthritis and Cartilage Degeneration: A Randomized, Double-blind, Multicenter, Placebo-Controlled Clinical Trial
Source: Pharm Res. 2025 Jan 28;42(2):249–69. doi: 10.1007/s11095-025-03818-2 (PMC11880083; doi:10.1007/s11095-025-03818-2)

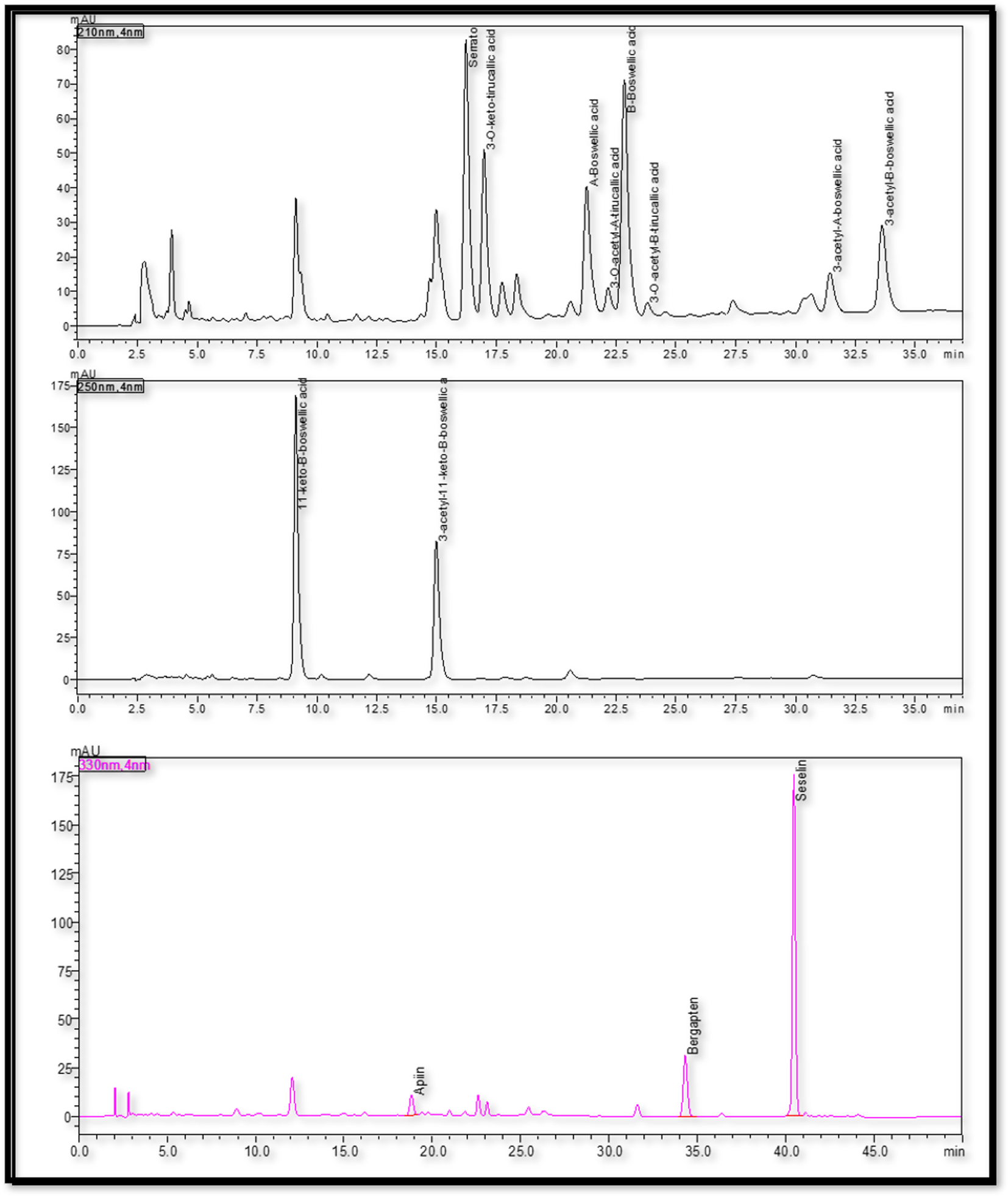

Supplement: Supplementary file 1 — Representation of the measured components of nutraceutical using HPLC-PDA (PNG 400 KB) [file 11095_2025_3818_Fig9_ESM.png]

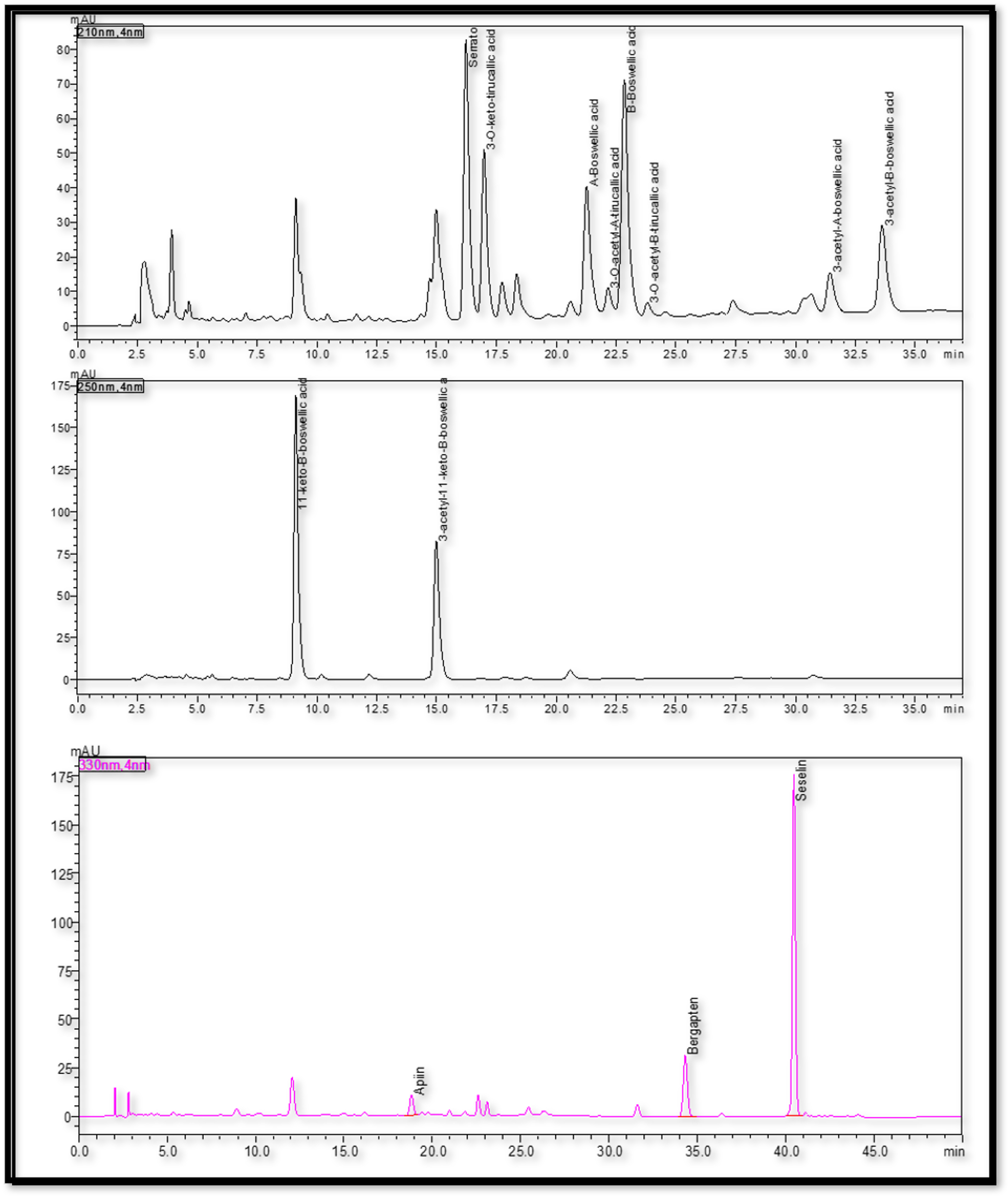

Supplement: Supplementary file 2 — High Resolution Image (TIF 1865 KB) [file 11095_2025_3818_MOESM1_ESM.tif]
